# Effects of intestinal flora on the pharmacokinetics and pharmacodynamics of aspirin in high-altitude hypoxia

**DOI:** 10.1371/journal.pone.0230197

**Published:** 2020-03-12

**Authors:** Yuemei Sun, Juanhong Zhang, Anpeng Zhao, Wenbin Li, Qiangsheng Feng, Rong Wang

**Affiliations:** 1 Key Laboratory for Prevention and Remediation of Plateau Environmental Damage, The 940th Hospital of Joint Logistics Support Force of Chinese People’s Liberation Army, Lanzhou, China; 2 School of Pharmacy, Lanzhou University, Lanzhou, China; University of Minnesota Twin Cities, UNITED STATES

## Abstract

Since hypobaric hypoxia significantly affects metabolic characteristics of intestinal flora, which plays an important role in the biotransformation of aspirin, high altitudes may influence the pharmacokinetics and therapeutic effects of aspirin in the intestines. In the present study, to test alterations of intestinal microbiota at high altitude comparing to that at low altitude, we analyzed rat feces from plain group and high-altitude group by 16S rRNA analysis. To detect concentrations of aspirin and salicylic acid, we established a reliable liquid chromatography tandem mass spectrometry method to measure aspirin and salicylic acid concentrations in fecal suspensions and plasma. Our study found that the plateau hypoxic environment caused a significant increase in *Bacteroides* in rat feces, while *Corynebacterium*, *Prevotella*, and *Coprococcus* were declined. In addition, compared with the plain group, the metabolic activity of fecal suspensions from the plateau group on aspirin was significantly reduced. More importantly, these changes in the intestinal microbiota led to increasing absorption of aspirin in the rats after rapidly ascent to the plateau, and a reduction in the pharmacodynamic index TXB2, which would possibly result in bleeding. In conclusion, our research provides new ideas for changes in plateau pharmacokinetics, and then guide the corresponding reduction in aspirin dose for the population quickly entering the plateau.

## Introduction

The gut flora is the most momentous and diverse microbial community that functions symbiotically with humans and animals[1]. These complex populations of bacteria, viruses and fungi are crucial not only for maintaining inherent health, but also in metabolizing exogenous xenobiotic[2]. It is worth mentioning that the ability of the intestinal flora to metabolize drugs is efficient but has often been underestimated in the past[3]. The gut microbiota is not static, but rather is highly variable. Consequently, any alteration or dysbiosis of the gut microbiota could directly or indirectly influence microbe-mediated bioactivation of the drug[4]. The hypoxic characteristics of the plateau environment have significant influences and constraints on the activities and behaviors of the living organisms, which can change the composition of intestinal microbes[5]. Furthermore, there is little research on the changes of pharmacokinetics mediated by intestinal flora under high-altitude hypoxia. This will provide new ideas for the changes of pharmacokinetics in plateau and better guidance for people who experienced acute exposure to hypoxia to use drugs reasonably.

Aspirin, one of the classic drugs absorbed through the gastrointestinal tract, which is widely used for primary prevention of cardiovascular diseases and secondary prevention after acute phase stabilization. Recently, we are highly concerned about the therapeutic status of aspirin, which may cause bleeding risk after long-term use. Before the aspirin is absorbed into the blood, the intestinal flora plays a considerable role in its biotransformation[6]. If the plateau environment changes the metabolic activity of the intestinal flora, then it could affect the pharmacokinetics and therapeutic outcome of aspirin. There is little knowledge about gut microbiota-mediated changes of pharmacokinetics and pharmacodynamics of aspirin in the plateau environment. Eventually this may lead to failure of the appropriate anti-platelet aggregation or other side effects.

We herein attempt to concretely investigate what changes will occur in the intestinal flora of rats in high-altitude hypoxia. As the number of people entering the plateau has gradually increased, the rational use of aspirin on the plateau should arouse our close attention. In particular, with the gut flora as the train of thought, we explored the results of aspirin treatment for changes in the intestinal flora mediated by the plateau environment.

## Materials and methods

### Materials

The aspirin (lot no. BJ34684) was purchased from Bayer Health Care Co. Ltd. (Germany). The standard substance of aspirin (lot no. 100113–201104) was obtained from China drug and biologic product standardization station (Beijing, PR China). The salicylic acid (lot no. J22S6J3607) was gained from Shanghai Yuan Ye Biological Technology Co. Ltd. Benzoic acid (lot no.100419-200301) was obtained from China Food and Drug Identification Research Institute. The Thromboxane B2 enzyme-linked immunosorbent assay (TXB2 ELISA) Kit (ADI-900-002) was gained from Enzo Life Sciences, Inc. Methanol and acetonitrile were using HPLC grade, available from Merck KGaA (Germany). Formic acid was chromatographed and purchased from Dima (USA). ANC VITEK2, GP VITEK2 and GN VITEK2 are all purchased in Merière. *Bacteroides vulqatus* and *Prevotella copri* are provided by the 940th Hospital Laboratory.

### Animal experiments

Healthy SPF-grade Wistar rats, weighing 180–200 g, were purchased from the Lanzhou Veterinary Research Institute and the certificate number (GAN) 2015–0001). The experiment was approved by the Ethics Committee of the 940th Hospital and was conducted in accordance with relevant ordinances and regulations. Rats were housed in rat box (475mm×345mm×200mm, six rats per cage) under controlled condition (temperature, 20–22°C, humidity, 50±10%), which was in line with hygiene standards. Rats were fed with standard SPF-grade diet and permitted water ad libitum. The rats were randomly assigned into two groups: plain group in Lanzhou in which the elevation is 1500m and acute plateau Group in the Plateau Field Animal Laboratory of Yushu Tibetan Autonomous Prefecture in which the elevation is 4100m (n = 12 for each). The two groups started the experiment at the same time point. The experiments in the plain and plateau are performed in standard laboratories, and the conditions of temperature and humidity are consistent. The difference is that the oxygen content of the plateau and plain is 12.9% and 21%, respectively.

### 16S rRNA analysis

The feces of the twelve rats in each group were divided into three small portions stored at −80°C until DNA extraction. Fecal samples were sent to the BGI gene for 16S rRNA analysis in order to discover alterations in intestinal flora after rapidly ascent to high altitude. Cetyltrimethyl Ammonium Bromide (CTAB) method for extracting genomic DNA from fecal samples, followed by PCR analysis immediately[5]. We conducted bioinformatics analysis on qualified samples. Paired-end sequencing through the Illumina platform (Hiseq or Miseq) removes low-quality reads and uses the software USEARCH (v7.0.1090) to cluster the stitched tags into OTUs. After obtaining the OTU representative sequence, the result was compared with the database for species annotation by Ribosomal Database Project (RDP) Classifier (v2.2) software, and the confidence threshold was set to 0.6. Sample species complexity and species difference analysis between groups based on OTU and species annotation results. We combined all species with abundance of species below 0.5% in all samples into others. Finally, we performed species annotation analysis and heat map analysis on each sample at the genus level.

### Extraction of faecal incubation matrix

The feces of each rat (approximately 0.1 g) was infiltrated in 0.9 mL of cold sodium chloride solution using scraper, and the system was centrifuged at 500 g for 5 min. The resulting supernatant was sonicated for 10 min, then centrifuged at 10,000g for 20 min. The supernatant was evaluated for the activity of intestinal microbes in the plateau through the metabolism of drugs[6].

### Analysis of aspirin metabolism in fecal suspensions

The reaction system (total volume 0.5mL) containing 0.1mL of 0.25mM aspirin, 0.1mL stool suspensions and 0.3mL of 0.1M phosphate buffer (pH7.0) incubated 12h, 24h and 36h at 37°C. The reaction was terminated by adding 570 μL frozen acetonitrile into the incubation system (30 μL). Each tube vortexed for 30 seconds, then centrifuged for 5 minutes at 13,000 rpm. The organic layer was transferred to a clean inner cannula, and 10 μL samples were inhaled into the ultra-fast liquid chromatography/tandem mass spectrometry (UFLC-MS/MS) system in order to analyze the aspirin and transformed salicylic acid.

### UFLC-MS/MS analysis

Aspirin and salicylic acid concentrations were analyzed using UFLC-MS / MS. High performance liquid chromatography (UFLC-20A, Shimadzu Corporation, Japan); Shim-pack XR-ODS column; Triple quadrupole mass spectrometer (API3200, Applied Biosystems, USA). The atomization temperature was 250°C and the flow rate is 0.4 mL/min[5]. Electrospray ionization assay was used for in vitro incubation concentration of remaining aspirin and plasma concentration of salicylic acid in vivo. The mobile phase was acetonitrile–water-formic acid (80:20:0.1, v/v/v). The detection ion pairs of aspirin, salicylic acid and internal standard benzoic acid were *m/z* 178.8→137.0, *m/z* 136.8→93.0, *m/z* 120.8→77.0, respectively, and the collision induced dissociation voltage was -11 psi, -26 psi and -24 psi, respectively. The collision energies are -13 eV, -26 eV, and -18 eV, respectively. The mass spectrometry spray voltage was -3500 V, and the atomization temperature was 250°C.

### Pharmacokinetic experiments

Rats were randomly divided into two groups, six in each group, which were plain group and plateau group. The drug administration of these two groups of rats is the same: overnight after fasting, each rat was given a dose of oral aspirin (31.5 mg/kg) dissolved in 2mL of sterilized water. The emphasis here was that aspirin has been removed enteric coatings with a spatula. Blood samples were gathered into heparinized tubes at 10 min, 20 min, 30 min, 45 min, 1 h, 1.25 h, 1.5 h, 2 h, 4 h, 6 h, 8 h, 12 h and 24 h after administration. The whole blood specimens were centrifuged at 5000 rpm for 5 min to obtain plasma and immediately stored at -20°C until quantitative analysis.

### Data analysis

The pharmacokinetic data of salicylic acid were analyzed by Software DAS 2.0. Statistical analysis was performed using SPSS version 22.0 software independent sample t-test.

## Results

### Methodological investigation

Impurities in the plasma and fecal incubation solution do not interfere with the determination of the sample, and have strong specificity. The retention time of salicylic acid in plasma was 1.49min. The standard curve equation is y = 0.0162x + 3.12, R^2^ = 0.9995. The salicylic acid concentration has a good linear relationship in the range of 100 ~ 40,000 ng / mL, with intra-day precision less than 7.46% and intra-day precision less than 6.06%. The recoveries at salicylic acid concentrations of 300, 25000, and 38000 ng / mL were 107.00%, 90.12%, and 104.82%, respectively. The stability of salicylic acid was measured at a plasma concentration of 25000 ng / mL. The RSD% when stored at room temperature for 24 hours, three times of repeated freeze-thaw, and stored at 4° C for one month were 6.92%, 7.21%, and 4.54%, respectively.

The standard curve equations of aspirin and salicylic acid peaks in stool samples are y = 0.092x + 1.82, R^2^ = 0.9994 and y = 0.016x + 1.39, R^2^ = 0.9992. Aspirin and salicylic acid have a good linear relationship in the concentration range of 100 ~ 40,000 ng / mL. The intra-day precision of aspirin is less than 8.01%, and the intra-day precision is less than 6.20%. The recoveries at aspirin concentrations of 125, 25000, and 38000 were 106.67%, 104.36%, and 102.01%, respectively. The stability of aspirin was measured at a plasma concentration of 25000ng / mL. The RSD% when stored at room temperature for 24h, three times of repeated freeze-thaw cycles, and stored at 4° C for one month were 5.08%, 3.18%, and 3.61%, respectively. The intra-day precision of salicylic acid is less than 7.35%, and the intra-day precision is less than 4.42%. When salicylic acid concentration was 125, 25000, 38000, the recoveries were 87.73%, 105.25%, and 104.82%, respectively. The stability of salicylic acid was measured at the plasma concentration of 25000ng / mL. The RSD% when stored at room temperature for 24h, three times of repeated freeze-thaw, and stored at 4°C for one month were 7.94%, 4.27%, and 2.64%, respectively.

### Effect of plateau hypoxia on intestinal flora in rats

**Fig 1:**
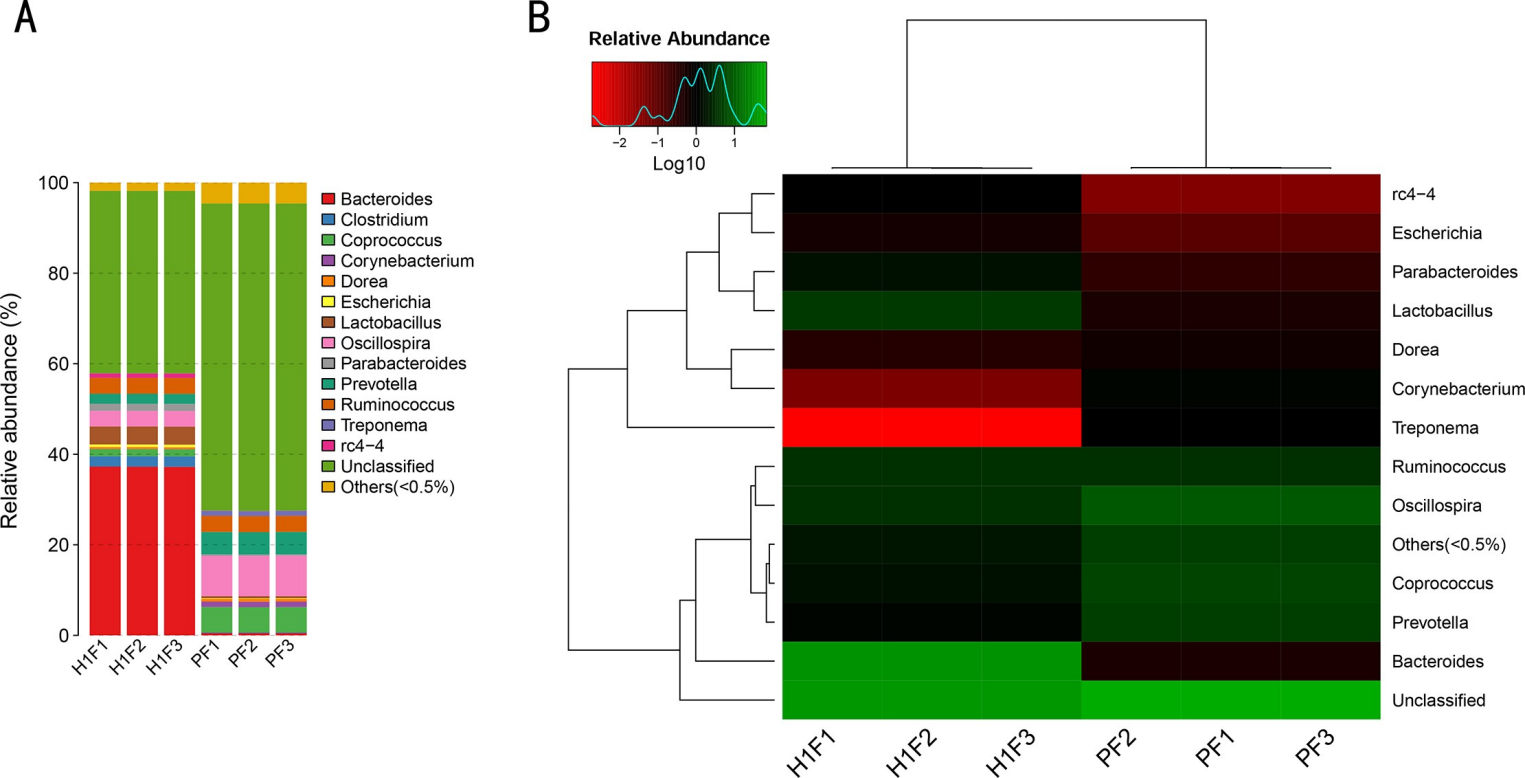
Changes of intestinal flora in rats (PF: Stool samples of plain group, H1F: Stool samples of high-altitude group). (A) Specimen profiling histogram based on OTU abundance, (B) Heatmap at genus level.

We thoroughly analyzed the composition of the intestinal flora of rats by 16S rRNA, and explored how the composition of the intestinal flora of rats after acute exposure to hypobaric hypoxia changed compared with the plain group. Specimen annotation analysis is a histogram of fecal samples, from which the proportion of different species in each sample can be visualized (Fig 1(A)). At the genus level, compared with the plain group, the plateau environment group *Bacteroides*, *Corynebacterium*, *Prevotella*, and *Coprococcus* showed significant changes. Heatmap is a graphical representation of the color gradients used to represent the magnitude of the values in the data matrix, and cluster analysis based on the abundance similarity of species or samples. The combination of clustering results and sampling environment grouping information can be used to know the clustering of samples in similar environments, and can directly reflect the differences and similarities of sample community composition. As shown in Fig 1(B), Longitudinal clustering represents a similar situation in which all species are expressed between different samples. The smaller the color distance, the more similar the species composition and abundance of the sample. Lateral clustering indicates that the species is similar in abundance in each sample. In combination with Fig (1A and 1B), at the genus level, the *Bacteroides* significantly increased, while *Corynebacterium*, *Prevotella*, and *Coprococcus* significantly decreased after rapid entry into the plateau. Species composition and abundance indicate that plateau hypoxia is an important factor affecting intestinal microbial homeostasis.

### Determination of aspirin concentration

**Fig 2:**
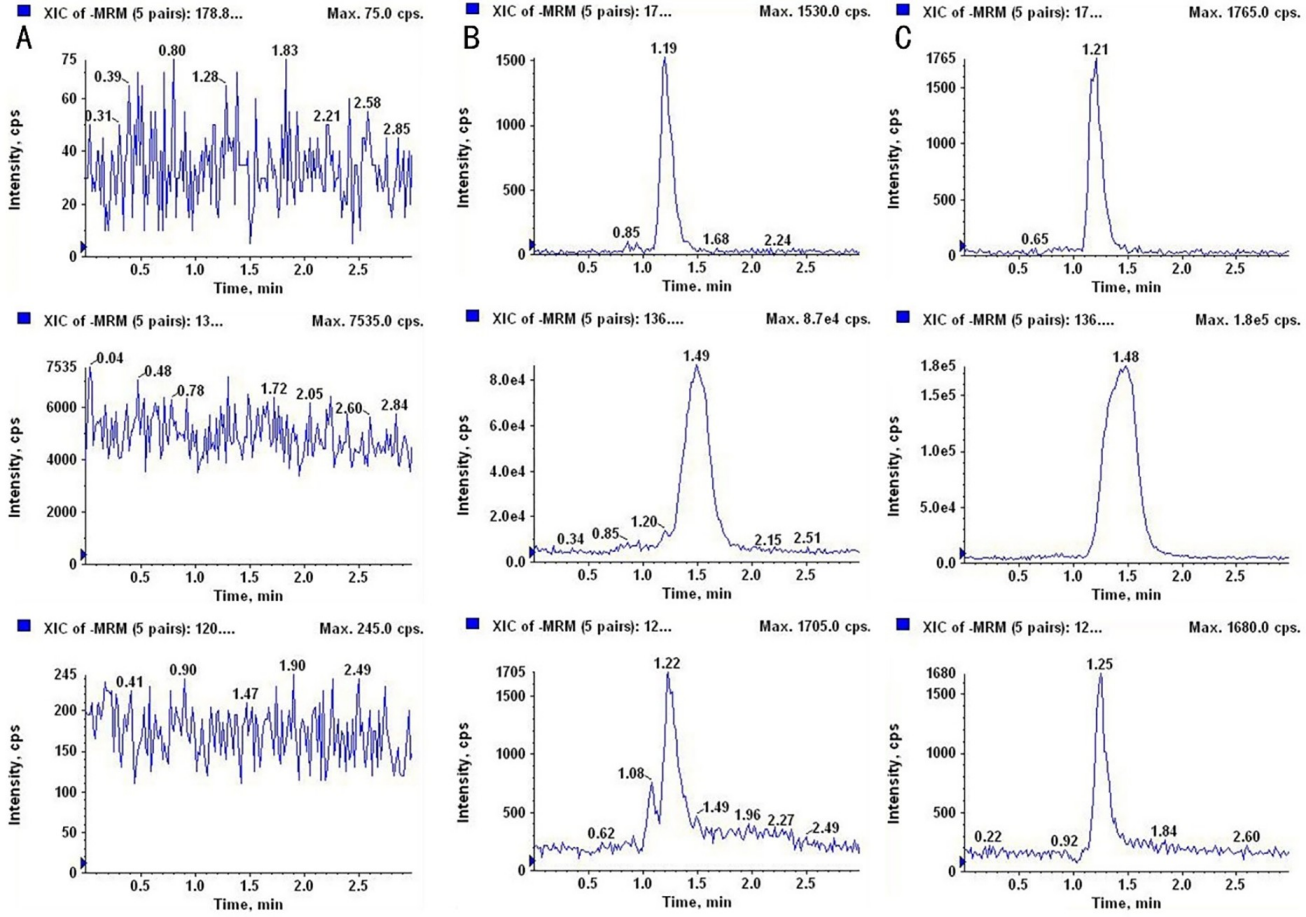
The chromatograms of aspirin and salicylic acid in rat stool samples. (A) blank fecal fluid, (B) blank fecal fluid spiked with standard aspirin and salicylic acid, (C) rat stool samples.

To determine the intestinal flora-mediated metabolism of aspirin at high altitudes, the metabolic activity of aspirin in stool samples was tested. Fecal samples from collected plain and high- altitude rats were tested to identify the metabolic characteristics and metabolic activity of aspirin in each group. As shown in the chromatograms of aspirin and salicylic acid in Fig (2A–2C), under our experimental conditions, the retention times of aspirin, salicylic acid and benzoic acid were 1.19, 1.49 and 1.22 min by our system, respectively. No impurity interference was seen near the peak time. Our results show that this method has good specificity and selectivity and can be well used for the analysis of stool samples.

### Determination of metabolic activity of aspirin

**Fig 3:**
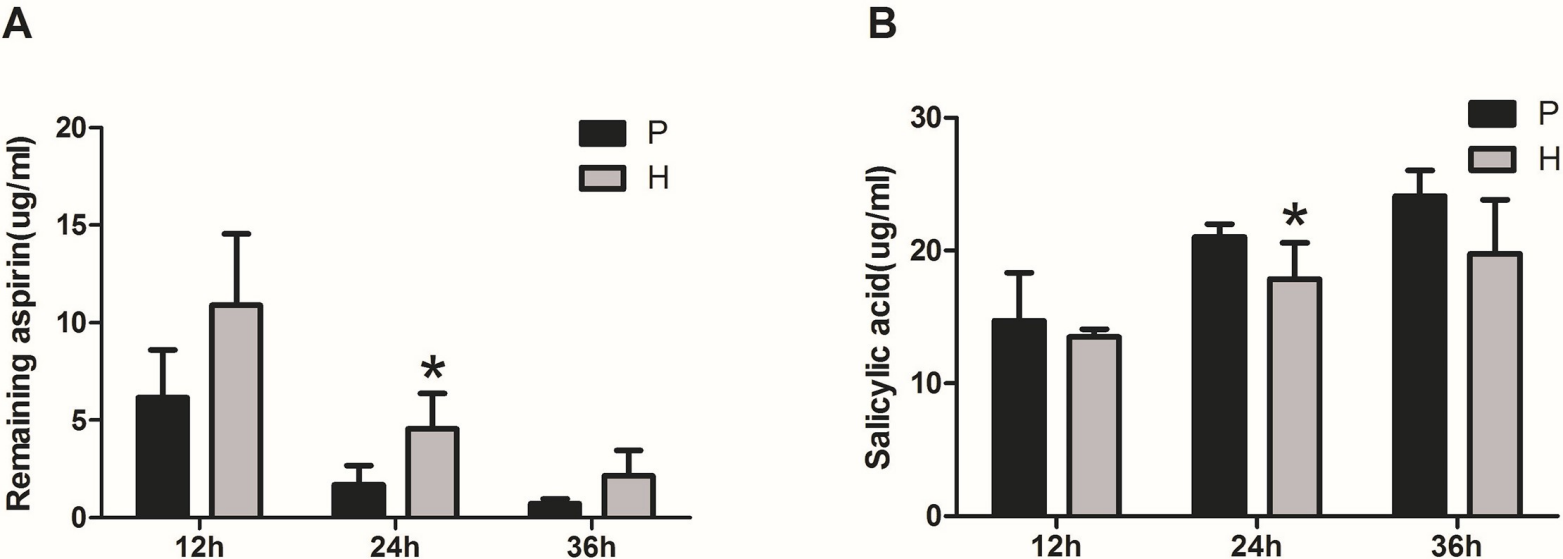
Aspirin-metabolizing activities in rat fecal samples. (P: plain group, H: high altitude group). (A) the remaining amount of aspirin; (B) formation of the salicylic acid. *p<0.05, compared with P.

To explore the metabolism of aspirin mediated by plateau hypoxic intestinal flora, aspirin was incubated with rat fecalase enzyme suspension for 12 h, 24 h and 36 h, and the remaining aspirin was quantified. As shown in Fig 3A, the residual amount of aspirin decreased over time, indicating that fecal microbial enzymes play a role in the metabolism of aspirin. Based on the measured data, after 12 h of incubation, the amount of aspirin in the plain group was 6161.67 ng/mL, and in the plateau group was 10893.33 ng/mL. After 24 h of incubation, the level of aspirin decreased by 95.12% and 86.84%, respectively, compared with their corresponding samples at 0 h, and there was a significant difference (*P* <0.05). In particular, this result indicates that the metabolic activity of aspirin is inhibited in the plateau environment, and it is also demonstrated that the intestinal flora is indeed involved in the metabolism of aspirin. Meanwhile, salicylic acid was discovered as a major metabolite of aspirin by fecal incubation and the formation of salicylic acid was detectable within 12 h of incubation. As the incubation time prolonged, as shown in Fig 3A, the amount of salicylic acid increased continuously and was positively correlated with the elimination of the maternal drug. In vitro incubation experiments have fully confirmed that changes in the metabolic characteristics of aspirin in high-altitude hypoxia are closely related to the inhibition of intestinal flora metabolism.

### Effects of plateau on the pharmacokinetics of aspirin

**Fig 4:**
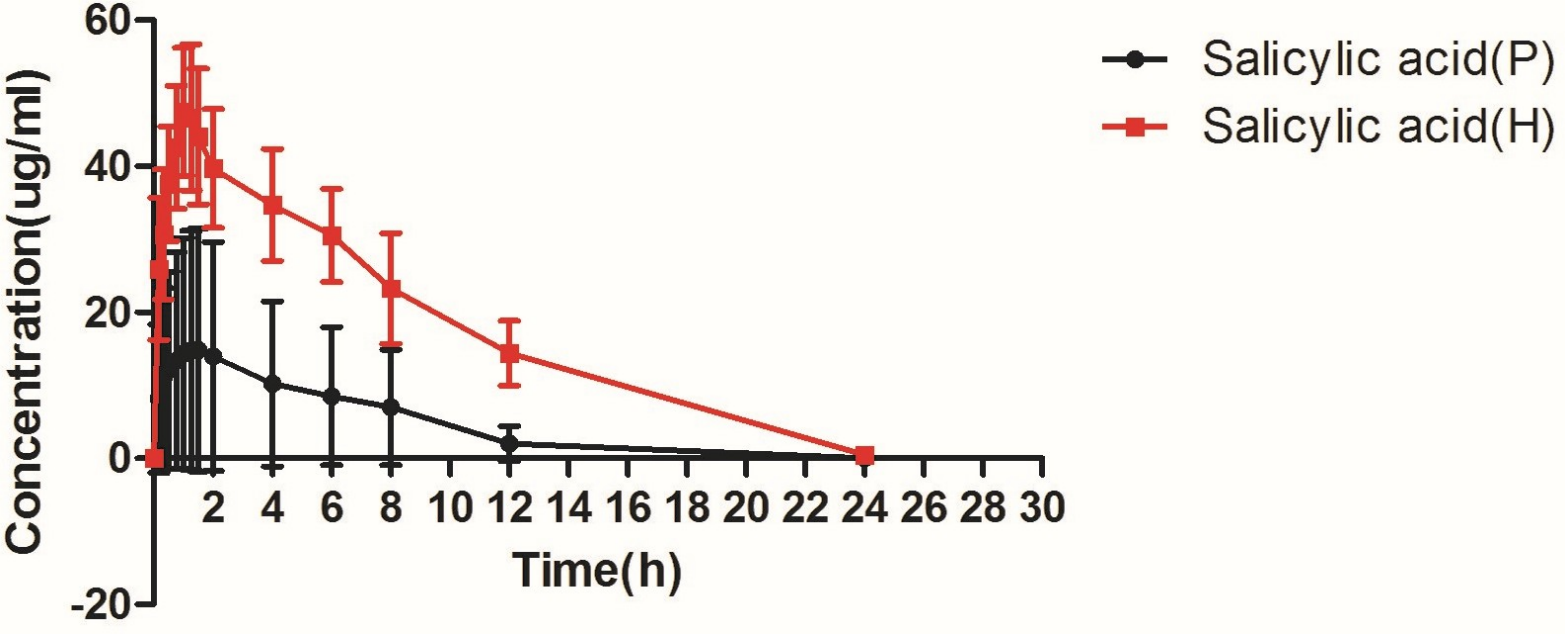
Mean plasma concentration–time curve of salicylic acid in rats (P: plain group, H: high altitude group).

Subsequently, in order to study the effects of plateau environment on the pharmacokinetic of aspirin in rats, the plasma levels of salicylic acid were determined after oral administration of aspirin in plain and plateau group rats. Only 25% of the oral dose of the drug was in the original form at 0.5 h after absorption, and the concentration of aspirin was lower for that orally administered aspirin is rapidly converted to salicylic acid in the body. In this experiment, the plasma concentrations of salicylic acid were measured to quantify the pharmacokinetic profile of the absorbed aspirin. The concentration-time curves in plasma obtained from the plain and plateau groups have semblable shapes which were depicted in Fig 4. The area under the concentration curve (AUC) and the maximum concentration (C_max_) obtained an increase in the plateau group, whereas plasma clearance (CL) decreased, compared with the plain group. As shown in Table 1, the AUC and the C_max_ were significantly increased 82.20% and 61.03%, respectively, and the CL was obviously reduced by 43.55%, compared with the plain group. Meanwhile, the AUC, C_max_ and CL value were statistically significant (*P<*0.01).

**Table 1:** Pharmacokinetic parameters of salicylic acid (P: plain group, H: high altitude group).

Parameter	P	H
AUC(0-t) (μg/L*h)	203923.450±30938.308	387754.442±116353.898**
AUC (0-∞) (μg/L*h)	255421.273±22391.054	465375.016±82929.292**
MRT(0-t) (h)	4.488±0.215	5.529±1.250
MRT (0-∞) (h)	7.597±1.388	8.452±1.710
t1/2z(h)	5.091±1.086	5.321±1.550
Tmax (h)	1.333±0.289	1.167±0.204
CLz/F(L/h/kg)	0.124±0.011	0.070±0.012**
Vz/F(L/kg)	0.918±0.263	0.544±0.214
Cmax(μg/L)	30533.333±4013.830	49166.667±8520.896**

**p<0.01 compared with P.

### Aspirin antiplatelet effect in the plateau

**Fig 5:**
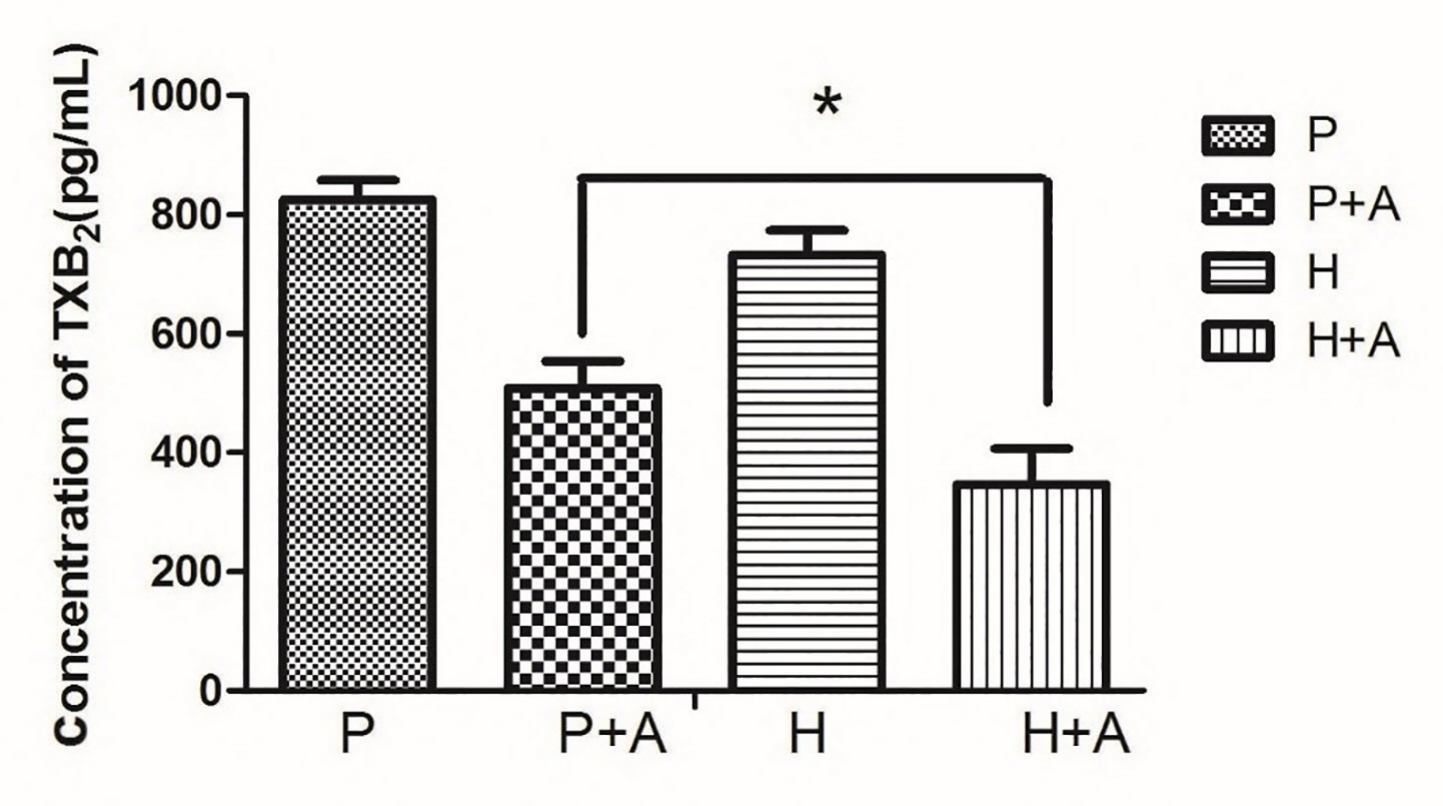
The content of TXB2 in plasma of rats (P: plain group, H: high altitude group, P+A: the plain group was given aspirin, H+A: the plateau group was given aspirin).

Next, we investigated whether the pharmacokinetic parameters of aspirin affect the therapeutic efficacy in the plateau. Low-dose aspirin produce antithrombotic effects by significantly reducing Thromboxane A_2_(TXA_2_) production. It is worth mentioning that TXA_2_ is immediately converted to TXB_2_ in vivo. In this experiment, the concentration of TXB_2_ in the plain and plateau groups after administration of aspirin was quantitatively analyzed by ELISA kit to clarify whether the therapeutic effect of aspirin will change in the plateau environment. According to the analysis program and statistical analysis of TXB_2_ ELISA kit, the results (Fig 5) showed that after the rats entered the plateau rapidly, compared with the plain blank group, the concentration of TXB_2_ in the plateau blank group decreased. Although there was no significant difference after aspirin treatment, TXB_2_ was significantly decreased by 31.73%, indicating that the plateau environment enhanced aspirin inhibition of platelet TXB_2_ production, which is consistent with the increase of aspirin absorption in the high-altitude environment. Therefore, the bleeding tendency of rats would increase, suggesting that we should closely monitor blood concentration when using aspirin in the plateau.

### The biotransformation of aspirin involves bacteria

**Fig 6:**
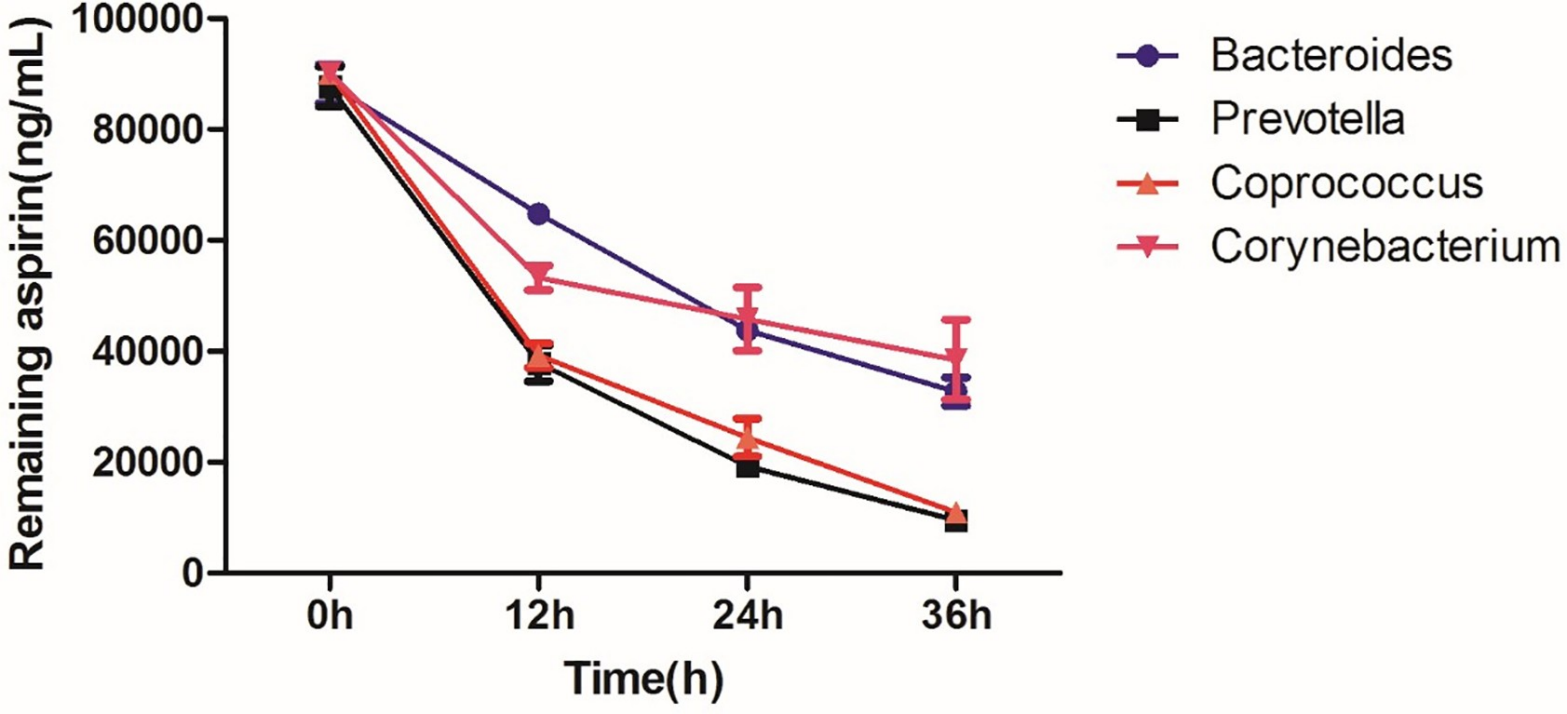
The bacteria involved were incubated with aspirin.

We attempted to elucidate the possible bacteria involved in changes of the pharmacokinetics and pharmacodynamics after aspirin treatment in the hypoxic environment. The rat feces were subjected to three-zone scribing for bacterial fractionation and the Merial automatic bacterial identification instrument to identify bacteria for preparing standard strains, and then the bacteria with significant differences in 16SrRNA were selected as *Corynebacterium amycolatum* and *Enterococcus faecalis*. Subsequently, aspirin was cultured with *Corynebacterium amycolatum*, *Enterococcus faecalis*, and acquired and *Bacteroides vulgatus* and *Prevotella copri*. The results showed that, as shown in Fig 6, the residual amount of aspirin decreased with the increase of time, and the metabolic clearance of aspirin by *Prevotella copri* and *Enterococcus faecalis* was more significant.

## Discussion

This study shows that the gut microbiota can act as an “invisible organ”, which plays a key role in the changes of the pharmacokinetics and therapeutic outcomes of aspirin in the high-altitude environment. First, the analysis of 16S rRNA showed that the plateau hypoxic environment is an important factor affecting the composition and quantity of microorganisms. The data displayed that the *Bacteroides*, *Corynebacterium*, *Prevotella*, *and Coprococcus* changed significantly at the genus level (Fig 1). Moreover, the number of *Bacteroides* was significantly increased, while the *Corynebacterium*, *Prevotella*, *and Coprococcus* showed a significant reduction. Therefore, in addition to the use of antibiotics and probiotics, high-altitude hypoxic environment is also a pivotal factor influencing the homeostasis of the intestinal flora.

It is now increasingly accepted that the gut microbiota could influence drug pharmacokinetics and correspondingly bioavailability, efficacy or adverse effects, although previous demonstration exhibited changes in metabolic capability of enterocytes and hepatocytes[7]. Aspirin is a clinical classic that has recently been pushed to the forefront. The latest findings from the European Conference on Cardiology (ESC) confirm the importance of aspirin in secondary prevention of cardiovascular and cerebrovascular diseases. In contrast, the role of aspirin in primary prevention of cardiovascular disease is controversial, and the risk of bleeding caused by it should be closely monitored in the clinic[8]. Most of aspirin is rapidly absorbed in the small intestine, however, in our current study, it has been proved that the intestinal flora plays a vital role in the metabolism of aspirin. First, we established a proprietary method to determine aspirin and salicylic acid in rat fecal samples (Fig 2). Next, we used the method established above to evaluate the effects of rat fecal suspensions on the metabolism of aspirin in different environments (Fig 3). We demonstrated that the metabolic activity of the plateau fecal suspensions on aspirin is significantly decreased compared with the plain group. *Prevotella copri and Enterococcus faecalis* play significant roles in the metabolism of aspirin (Fig 6). The abundance of the *Prevotella*/*Bacteroides* may be beneficial for metabolism[9]. This may explain that the metabolic activity of aspirin in the high-altitude environment is inhibited. Aspirin is rapidly decomposed into salicylic acid in the blood and gastrointestinal tract after drug administration. At present, the pharmacokinetic study of aspirin is often quantified by salicylic acid[10]. Subsequently, we demonstrated that the hypoxic environment of the plateau increases the bioavailability of aspirin by attenuating the metabolic activity of the gut flora (Fig 4), which is consistent with the metabolic effects of fecal suspensions on aspirin. Aspirin can significantly reduce TXA_2_ at low doses without significant effect on PGI2 levels, resulting in an antithrombotic effect. Our results suggested that after the rats enter the plateau, it is necessary to reduce the amount of aspirin to prevent bleeding and the side effects of salicylic acid on the gastrointestinal tract (Fig 5).

To date, effects of gut microbiota on drug pharmacokinetics have been extensively studied. The microbiota could influence drug pharmacokinetics through selected mechanisms including prodrug activation[11], alteration of drug absorption[12], enterohepatic circulation of drugs[13], drug inactivation[14], and alteration of host drug metabolism[15]. However, the relationship between gut microbiota and host were extremely complex, since the influence of gut microbiota on drug metabolism is still explored and now being advanced by molecular biology such as metagenomics, metabolomics as well[7]. Our research revealed that the plateau environment has changed the composition and quantity of the intestinal flora and microbe-mediated drug pharmacokinetics, leading to a major re-evaluation of role of the gut microbiota in plateau pharmacokinetics. Therefore, we should pay attention to drugs metabolized by the intestinal flora in high-altitude environments, and if necessary, it is recommended to reduce the dose of aspirin according to clinical monitoring results at high altitudes.

## Conclusions

In summary, our results demonstrated that plateau hypoxia can regulate the pharmacokinetics and pharmacodynamics of aspirin by altering the metabolic activity of the intestinal flora. To the best of our knowledge, this is the first report that the metabolic activity of the intestinal flora caused by the high-altitude environment has been weakened and changed the bioavailability of aspirin. Further, the tendency to use aspirin is more likely to cause bleeding, which should arouse a high degree of concern for people rapidly exposure by plateau. This new finding provides a theoretical basis for the rational use of aspirin while quickly entering the plateau.
